# Supplementary material for: ARID1A governs the silencing of sex-linked transcription during male meiosis in the mouse
Source: eLife. 2024 Nov 26;12:RP88024. doi: 10.7554/eLife.88024 (PMC11594533; doi:10.7554/eLife.88024)
Supplement: Figure 2—source data 1. — There is no significant difference in the numbers of undifferentiated spermatogonia expressing PLZF in 1-month-old Arid1a cKO relative to Arid1afl/fl males. [file elife-88024-fig2-data1.docx]

Figure 2-Source Data 1: Wild-type and Mutant PLZF Positive Spermatogonia

| Section # | Number of PLZF+ spermatogonia | #Number of tubules examined | PLZF+  Spg/tubule | Average | SEM |
| --- | --- | --- | --- | --- | --- |
| WT1 | 648 | 91 | 7.12 | 8.87 | 1.33 |
| WT2 | 832 | 104 | 8 |  |  |
| WT3 | 782 | 68 | 11.5 |  |  |
| KO1 | 1005 | 91 | 11.04 | 10.45 | 0.49 |
| KO2 | 728 | 80 | 9.1 |  |  |
| KO3 | 883 | 82 | 10.76 |  |  |
| KO4 | 415 | 43 | 9.65 |  |  |
| KO5 | 149 | 12 | 12.41 |  |  |
| KO6 | 243 | 25 | 9.72 |  |  |

No significant difference in the numbers of undifferentiated spermatogonia expressing PLZF in 1-month-old *Arid1a*c KO *relative to Arid1a*^fl/fl^ males. SEM: Standard error of measurement.
