# Supplementary figures and images for: ARID1A governs the silencing of sex-linked transcription during male meiosis in the mouse
Source: eLife. 2024 Nov 26;12:RP88024. doi: 10.7554/eLife.88024 (PMC11594533; doi:10.7554/eLife.88024)

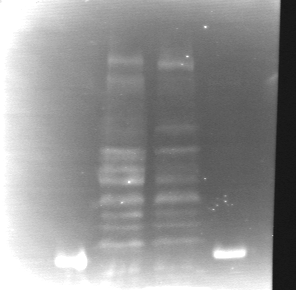

Supplement: Figure 2—figure supplement 1—source data 1. [file elife-88024-fig2-figsupp1-data1.tif]

Fig. S2A full Western blots

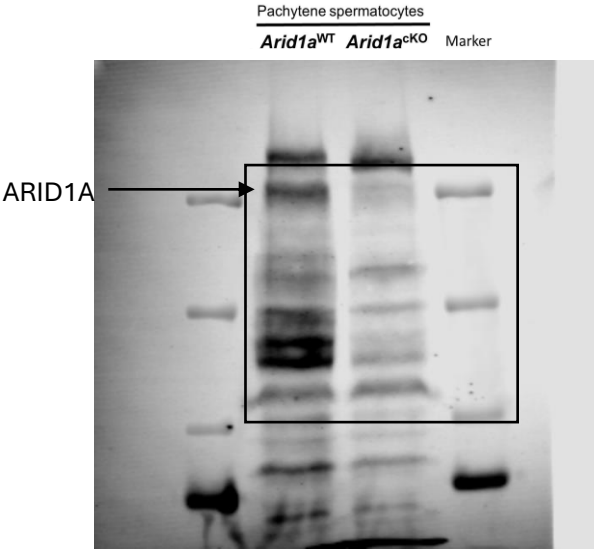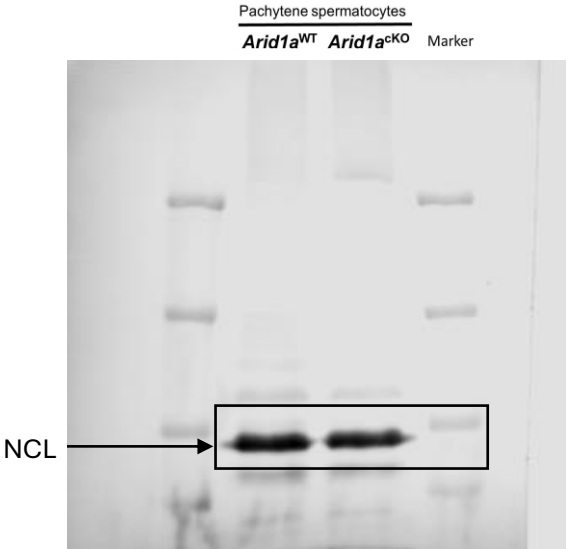

Supplement: Figure 2—figure supplement 1—source data 2. [file elife-88024-fig2-figsupp1-data2.zip › Figure 2-Figure Supplemental 1-source data 2.pdf]

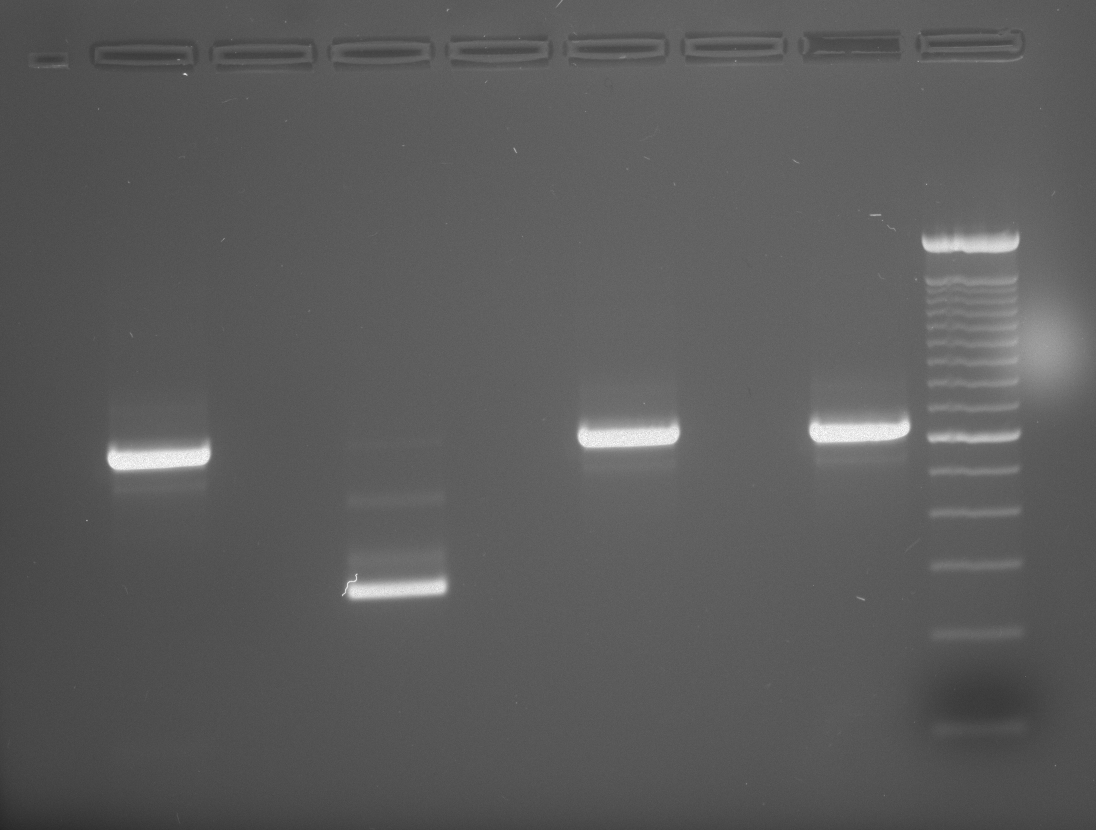

Supplement: Figure 2—figure supplement 3—source data 1. [file elife-88024-fig2-figsupp3-data1.zip › Figure 2-Figure Supplement 3-Source Data 1/Supplement Figure 4A raw source data.TIF]

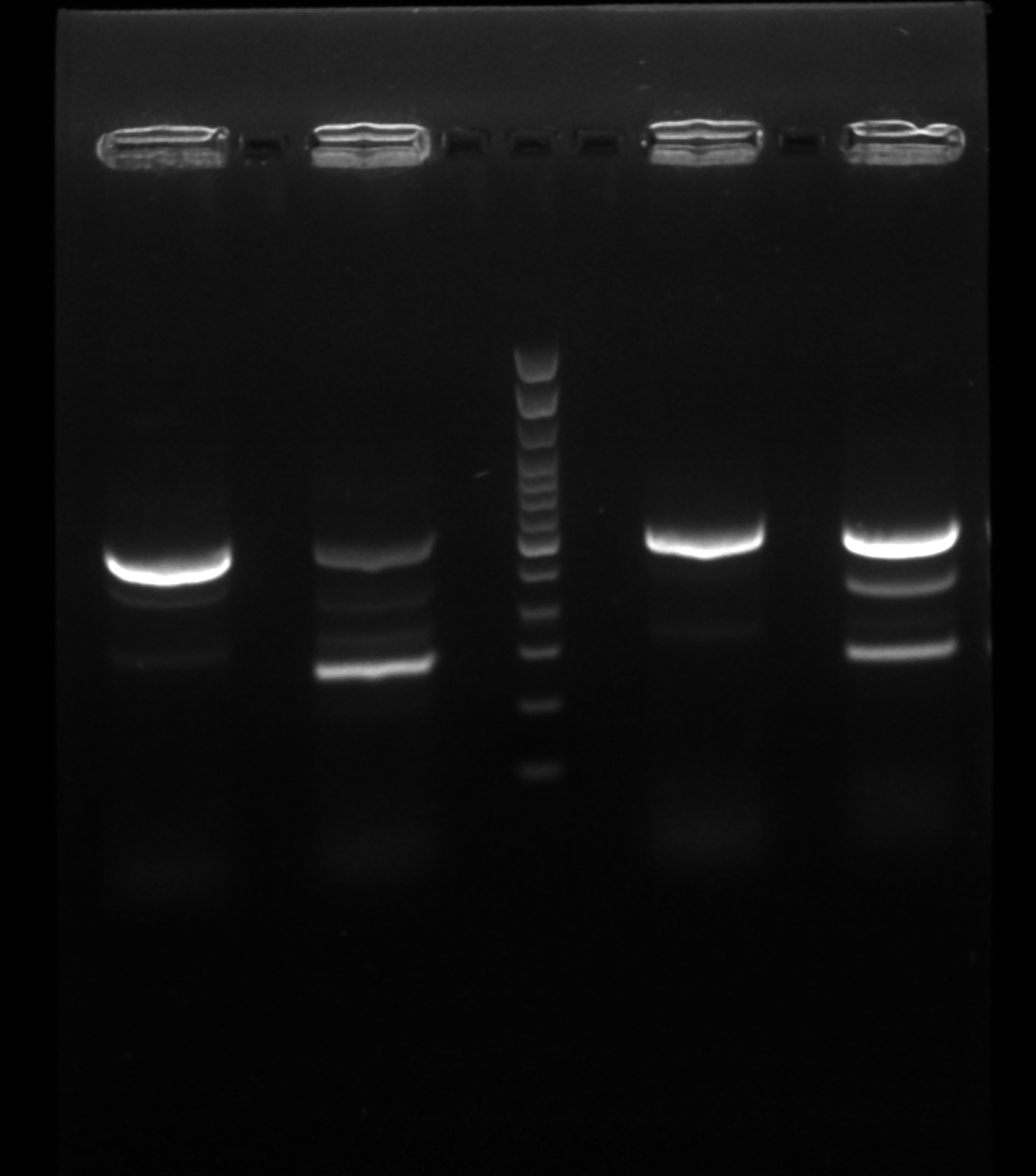

Supplement: Figure 2—figure supplement 3—source data 1. [file elife-88024-fig2-figsupp3-data1.zip › Figure 2-Figure Supplement 3-Source Data 1/Figure 2-Figure Supplement 3-Source Data 1.tif]

Figure 2-Figure Supplement 3-Source Data 2

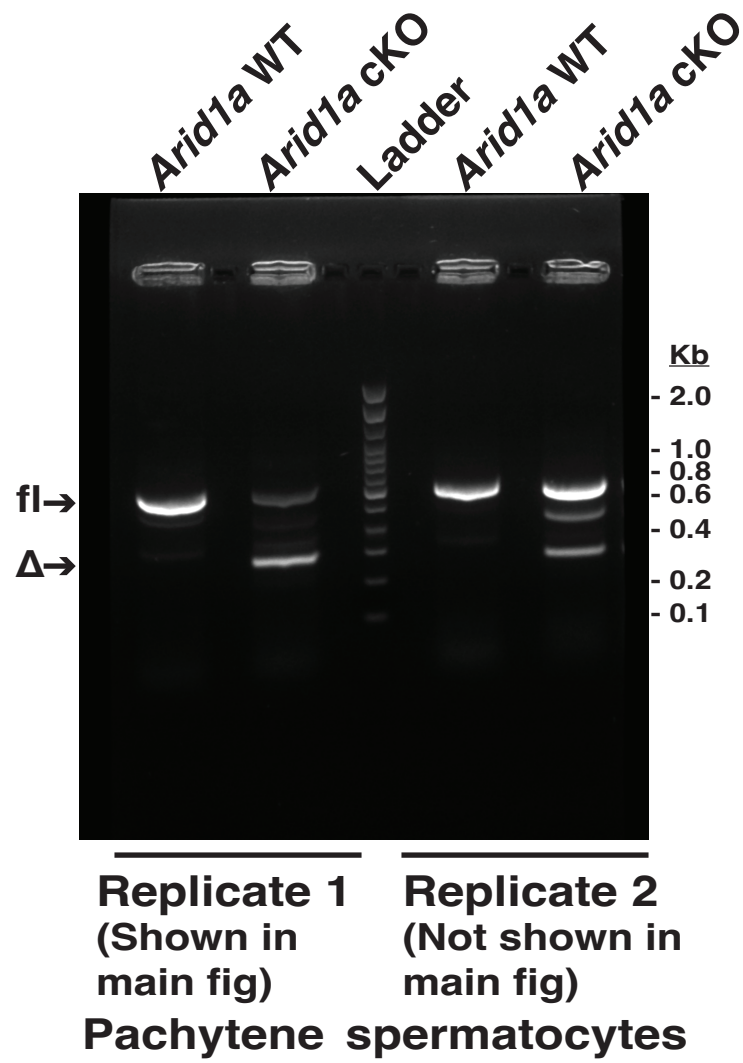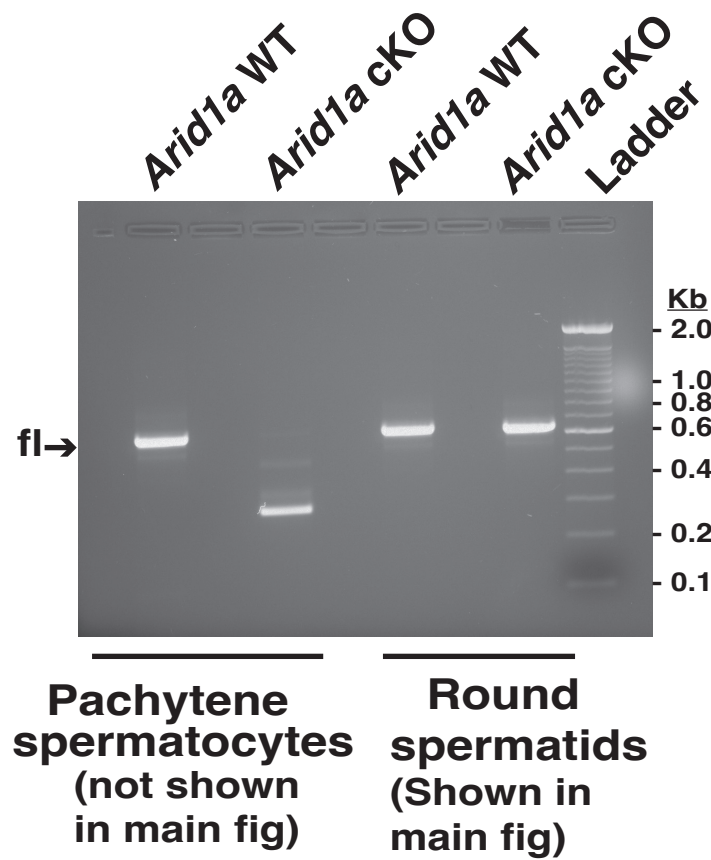

Supplement: Figure 2—figure supplement 3—source data 2. [file elife-88024-fig2-figsupp3-data2.zip › Figure 2-Supplement Figure 3a-raw source data 2.pdf]

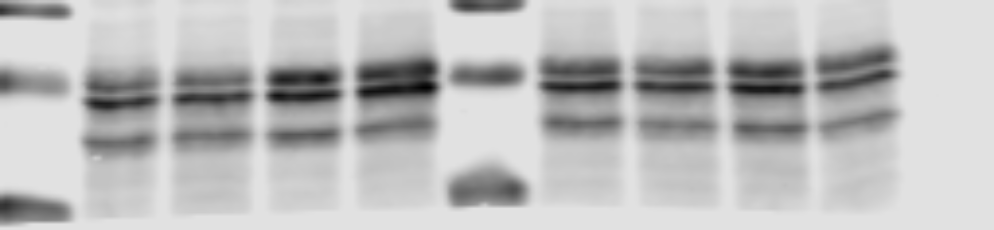

Supplement: Figure 5—figure supplement 1—source data 1. [file elife-88024-fig5-figsupp1-data1.zip › Figure 5-Figure Supplement 1-Source Data 1/Figure 5-Figure Supplement 1-source data- bottom 2.tif]

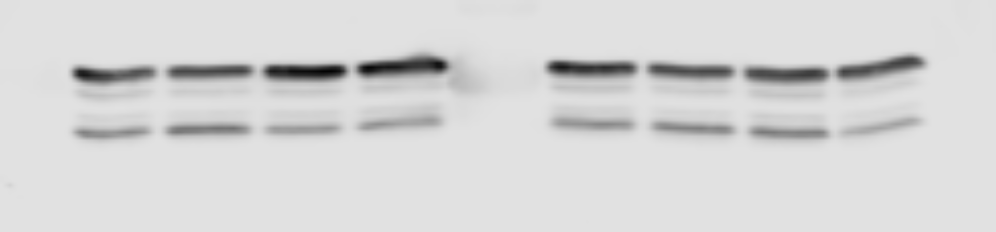

Supplement: Figure 5—figure supplement 1—source data 1. [file elife-88024-fig5-figsupp1-data1.zip › Figure 5-Figure Supplement 1-Source Data 1/Figure 5-Figure Supplement 1-source data- top 1.tif]
