# Supplementary material for: ARID1A governs the silencing of sex-linked transcription during male meiosis in the mouse
Source: eLife. 2024 Nov 26;12:RP88024. doi: 10.7554/eLife.88024 (PMC11594533; doi:10.7554/eLife.88024)
Supplement: Figure 5—figure supplement 1—source data 2. [file elife-88024-fig5-figsupp1-data2.zip › Figure 5-Figure Supplement 1-raw source data labeled 3.pdf]

Figure 5-Figure Supplement 1-raw source data labeled 3.pdf

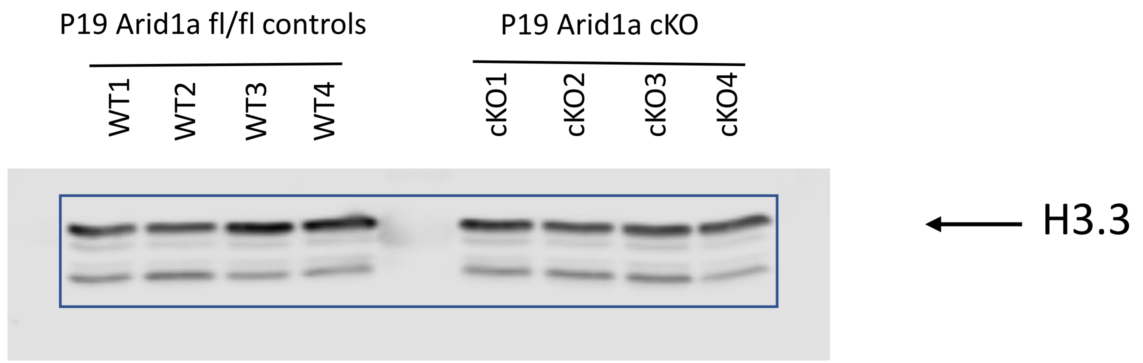

File name: Figure 5-Figure Supplement 1-raw source data labeled 3_Panel-1_H3.3Blot

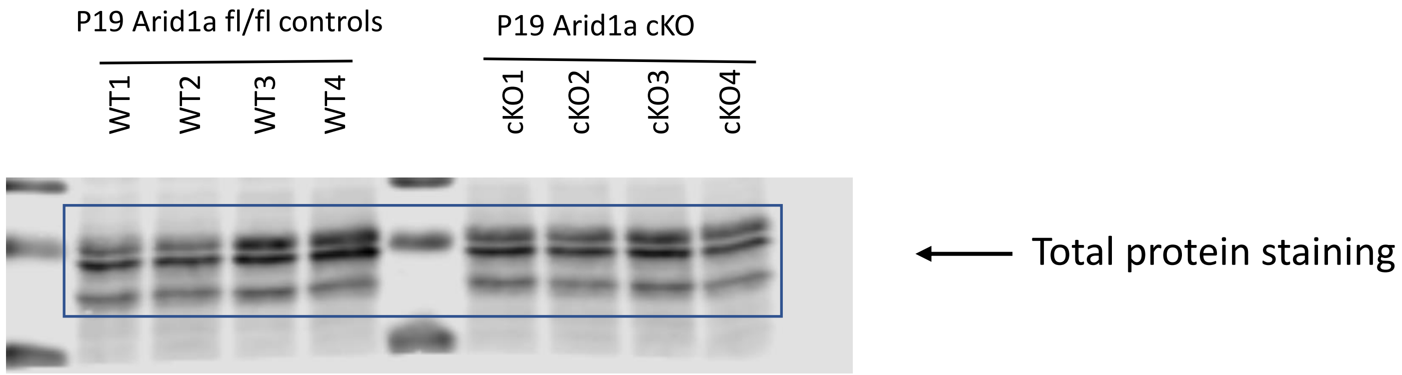

File name: Figure 5-Figure Supplement 1-raw source data labeled 3_Panel-2_total_protein_H3.3Blot
